# Supplementary material for: RESCRIPt: Reproducible sequence taxonomy reference database management
Source: PLoS Comput Biol. 2021 Nov 8;17(11):e1009581. doi: 10.1371/journal.pcbi.1009581 (PMC8601625; doi:10.1371/journal.pcbi.1009581)
Supplement: S1 Text — Methods and results summary on how database preparation affects taxonomic classification of real-world biological data from the Earth Microbiome Project. (DOCX) [file pcbi.1009581.s001.docx]

Supplemental Results

The Earth Microbiome Project (EMP) [[1]](https://paperpile.com/c/wmsNjO/DJ3su) was selected as a test dataset for comparing classification results, because it represents a broad cross-section of global microbial biodiversity. A representative subset of pre-processed 16S rRNA gene V4 sequences from 2000 samples from 15 different environmental sample types (empo_3 types) was downloaded from the EMP database (filename: "emp.150.min25.deblur.seq.subset_2k"). These sequences were filtered to keep only those detected at least 100 times and in at least 10 samples, in order to focus on the most abundant species. These sequences were classified using Naive Bayes classifiers trained on the SILVA, Greengenes, GTDB, or NCBI-RefSeqs sequences (trimmed to the V4 domain) using QIIME 2’s q2-feature-classifier plugin [[2]](https://paperpile.com/c/wmsNjO/1K9R2). RESCRIPt’s evaluate-taxonomy action was used to compare how classification depth compared for each classifier, measuring number of unique taxa identified, taxonomic label entropy, and the depth of classification (counting unannotated ranks, e.g., 'g__', as unclassified) (S1 Fig). Family-level taxonomic abundance predicted by each classifier is shown in Fig 5.

The impacts of database filtering on classification of real biological data was tested using the EMP data, as above, to demonstrate performance in diverse sample types (S2 Fig). Only a small effect was observed: whereas classification with the raw database sequences yields a species-level classification rate of 44.6%, classification with the filtered database (applying all filters described above, except for removing sequences with ambiguous genus or species labels) yields 45.1%, and applying a strict sequence filter (removing any sequence with one or more ambiguous base) yields 48.6% species-level classification of the EMP query sequences (S2 Fig). Removing sequences with ambiguous genus and species labels, on the other hand, led to a much lower level of classification at species level (35.3%) and even at lower ranks, and hence clearly leads to diminished usability, consistent with the benchmarks above. Of all EMP sequences classified, 95.1% were assigned the same taxonomic affiliation by all classifiers, and this did not lead to appreciable differences in average family-level taxonomic classification of different EMP sample types (data not shown). These results suggest that database filtering to remove ambiguous sequences (from a large database like SILVA) does not substantially alter classification results on real data, but trimming out low-quality sequences to reduce database size will nevertheless improve performance (e.g., reduce runtime and memory overheads for taxonomic classification [[2]](https://paperpile.com/c/wmsNjO/1K9R2)).

**References**:

1. [Thompson LR, Sanders JG, McDonald D, Amir A, Ladau J, Locey KJ, et al. A communal catalogue reveals Earth’s multiscale microbial diversity. Nature. 2017;551: 457–463.](http://paperpile.com/b/wmsNjO/DJ3su)

2. [Bokulich NA, Kaehler BD, Rideout JR, Dillon M, Bolyen E, Knight R, et al. Optimizing taxonomic classification of marker-gene amplicon sequences with QIIME 2’s q2-feature-classifier plugin. Microbiome. 2018;6: 90.](http://paperpile.com/b/wmsNjO/1K9R2)
