# Supplementary material for: RESCRIPt: Reproducible sequence taxonomy reference database management
Source: PLoS Comput Biol. 2021 Nov 8;17(11):e1009581. doi: 10.1371/journal.pcbi.1009581 (PMC8601625; doi:10.1371/journal.pcbi.1009581)
Supplement: S1 File — A jupyter notebook version is available at https://github.com/bokulich-lab/db-benchmarks-2020. (PDF) [file pcbi.1009581.s004.pdf]

# Making a 12S rRNA marker gene reference database.

The 12S rRNA marker gene is commonly used in encironmental DNA (eDNA) surveys in various ways, from studying the diets of feral swine (Anderson *et al*. 2018), through the study of fish (Milan *et al*. 2020).

Below is an example of how RESCRIPt, can make the process of making a custimized reference database much less onerous.

## Fetch 12S rRNA gene data

To keep the example short and sweet, we'll use RESCRIPt's `get-ncbi-data` to fetch only metazoan sequences. More information can be found here.

We'll also be sure to exclude a variety of potentially unhelpful reference sequences. We'll do all of this by using the following entrez search term: `txid33208[ORGN] AND (12S[Title] OR 12S ribosomal RNA[Title] OR 12S rRNA[Title]) AND (mitochondrion[Filter] OR plastid[Filter]) NOT environmental sample[Title] NOT environmental samples[Title] NOT environmental[Title] NOT uncultured[Title] NOT unclassified[Title] NOT unidentified[Title] NOT unverified[Title]`

Finally, we'll specify a list of taxonomic ranks that we'd like to extract for each sequence we download from GenBank. To make sure that we have a value for each rank, we'll eneable `--p-rank-propagation`. See the note on rank propagation within the RESCRIPt SILVA tutorial.

In [5]:

```
! qiime rescript get-ncbi-data \
    --p-query "txid33208[ORGN] AND (12S[Title] OR 12S ribosomal RNA[Title] OR 12
    --p-ranks  superkingdom kingdom subkingdom superphylum phylum subphylum supe
    --p-rank-propagation \
    --p-n-jobs 4  \
    --o-sequences metazoan-12S-ref-seqs.qza \
    --o-taxonomy metazoan-12S-ref-tax.qza \
    --verbose
```

```
Saved FeatureData[Sequence] to: metazoan-12S-ref-seqs.qza
Saved FeatureData[Taxonomy] to: metazoan-12S-ref-tax.qza
```

## Dereplicate data

Sequence repositories like GenBank, and others, often contain quite a bit of redundant data. For example, different research groups may generate sequence data for the same set of taxa with similar approaches (*i.e.* identical primers). We'll dereplicate the sequence data to reduce the size of our database, which will also reduce the time spent on all other downstream database curational steps.

```
! qiime rescript dereplicate \
    --i-sequences metazoan-12S-ref-seqs.qza \
    --i-taxa metazoan-12S-ref-tax.qza \
    --p-mode 'uniq' \
    --p-threads 4 \
    --p-rank-handles 'disable' \
    --o-dereplicated-sequences metazoan-12S-ref-seqs-derep.qza \
    --o-dereplicated-taxa metazoan-12S-ref-tax-derep.qza
```

```
Saved FeatureData[Sequence] to: metazoan-12S-ref-seqs-derep.qza
Saved FeatureData[Taxonomy] to: metazoan-12S-ref-tax-derep.qza
```

# Filter low-quality sequences

It's quite common for sequence repositories like GenBank to contain sequence data with ambiguous IUPAC nucleotides like `N`, `R`, `Y`, `M` ..., etc. We can remove such sequences with the `cull-seqs` action.

In [7]:

```
! qiime rescript cull-seqs \
    --i-sequences metazoan-12S-ref-seqs-derep.qza \
    --p-n-jobs 4 \
    --p-num-degenerates 5 \
    --p-homopolymer-length 8 \
    --o-clean-sequences metazoan-12S-ref-seqs-cull.qza
```

```
Saved FeatureData[Sequence] to: metazoan-12S-ref-seqs-cull.qza
```

# Filter by sequence length

The sequences we find on GenBank may be too short (or too long) for our needs. We can remove such sequences, for our purposes here. We'll make use of RESCRIPt's `filter-seqs-length` action. *Note, if you are interested in length trimming based on taxonomy you can use `filter-seqs-length-by-taxon`.*

Most metazoan 12S rRNA sequences range from 800-1000 bp Yang *et al*. 2014. However, many researchers appear to sequence ~ 200-400 bp of this gene. Thus, we'll exclude any sequences less than 200 bp. We'll also exclude spuriously long sequences (~1200 bp) too.

In [9]:

```
! qiime rescript filter-seqs-length \
    --i-sequences metazoan-12S-ref-seqs-cull.qza \
    --p-global-min 200 \
    --p-global-max 1200 \
    --o-filtered-seqs metazoan-12S-ref-seqs-keep.qza \
    --o-discarded-seqs metazoan-12S-ref-seqs-discard.qza
```

```
Saved FeatureData[Sequence] to: metazoan-12S-ref-seqs-keep.qza
Saved FeatureData[Sequence] to: metazoan-12S-ref-seqs-discard.qza
```

# Let's take a look at what we've got!

In [14]:

```
# filter taxonomy file to match that of the sequence file
```

```
    --i-taxonomy metazoan-12S-ref-tax.qza \
    --m-ids-to-keep-file metazoan-12S-ref-seqs-keep.qza \
    --o-filtered-taxonomy metazoan-12S-ref-tax-keep.qza
```

```
Saved FeatureData[Taxonomy] to: metazoan-12S-ref-tax-keep.qza
```

In [15]:

```
! qiime rescript evaluate-taxonomy \
    --i-taxonomies metazoan-12S-ref-tax-keep.qza \
    --o-taxonomy-stats metazoan-12S-ref-tax-keep-eval.qzv
```

```
Saved Visualization to: metazoan-12S-ref-tax-keep-eval.qzv
```

In [16]:

```
! qiime metadata tabulate \
    --m-input-file metazoan-12S-ref-tax-keep.qza \
    --o-visualization metazoan-12S-ref-tax-keep.qzv
```

```
Saved Visualization to: metazoan-12S-ref-tax-keep.qzv
```

In [18]:

```
! qiime rescript evaluate-seqs \
    --i-sequences metazoan-12S-ref-seqs-keep.qza \
    --p-kmer-lengths 32 16 8 \
    --o-visualization metazoan-12S-ref-seqs-keep-eval.qzv
```

```
Saved Visualization to: metazoan-12S-ref-seqs-keep-eval.qzv
```

As you can see, after all of our processing we have over 70k 12S rRNA reference sequences. We've also generated a few basic descriptors of these references too. Let's take these and build a classifier, and evaluate it.

# Build and evaluate our classifier

We are now ready to construct a classifier for our 12S rRNA reference sequences. We'll use the `evaluate-fit-classifier`, as this will not only make our classifier just like `qiime feature-classifier fit-classifier-naive-bayes`, but will also provide an evaluation of our "best-case estimate" of accuracy (*i.e.*, when all query sequences have one or more known matches within our reference database. See our other tutorials for more details.

In [20]:

```
! qiime rescript evaluate-fit-classifier \
    --i-sequences metazoan-12S-ref-seqs-keep.qza \
    --i-taxonomy metazoan-12S-ref-tax-keep.qza \
    --p-n-jobs 2 \
    --o-classifier ncbi-12S-metazoan-refseqs-classifier.qza \
    --o-evaluation ncbi-12S-metazoan-refseqs-classifier-evaluation.qzv \
    --o-observed-taxonomy ncbi-12S-metazoan-refseqs-predicted-taxonomy.qza
```

```
Saved TaxonomicClassifier to: ncbi-12S-metazoan-refseqs-classifier.qza
Saved Visualization to: ncbi-12S-metazoan-refseqs-classifier-evaluation.qzv
Saved FeatureData[Taxonomy] to: ncbi-12S-metazoan-refseqs-predicted-taxonomy.qza
```

In [22]:

```
! qiime rescript evaluate-taxonomy \
  --i-taxonomies metazoan-12S-ref-tax-keep.qza ncbi-12S-metazoan-refseqs-predict
  --p-labels ref-taxonomy predicted-taxonomy \
  --o-taxonomy-stats ref-taxonomy-evaluation.qzv
```

```
Saved Visualization to: ref-taxonomy-evaluation.qzv
```

In [ ]:

```
```
